# Reviewed article: Scharf A, Paulo DP, Paes GF, Cunha WL, Azevedo D, Guimarães B, Rodrigues N. Increased maternal and neonatal morbidity and mortality due to severe acute respiratory syndrome in the early years of COVID-19: a descriptive-analytical study, Rio de Janeiro state, Brazil, 2018-2021. Epidemiol Serv Saude 2025;34:e20240665

**DOI:** 10.1590/S2237-96222025v34e20240665.a

**Published:** 2025-08-11

**Authors:** Vicente Sperb Antonello

**Affiliations:** 1 Hospital Fêmina, Porto Alegre, RS, Brazil Hospital Fêmina Porto Alegre RS Brazil

## Peer review A

## Questionnaire

Does the manuscript contain new and significant information to justify publication? No

Does the Abstract (Summary) clearly and accurately describe the content of the article? Yes

Is the problem significant and concisely stated? No

Are the methods described comprehensively? No

Are the interpretations and conclusions justified by the results? No

Is adequate reference made to other work in the field? No 

## Manuscript Structure

Length of article is: Too short

Number of tables is: Too few

Number of figures is: Too few

**Please state any conflict(s) of interest that you have in relation to the review of this paper (state “none” if this is not applicable).**

None

**Interest:** Excellent

**Quality**: Below Average

**Originality:** Good

**Overall:** Below Avarage

**Recommendation:** Reject

## Comments

Aumento da morbimortalidade materna e neonatal por SRAG nos primeiros anos de COVID-19 nas nove Regiões de Saúde do Estado do Rio de Janeiro

Resumo: Embora as informações sejam claras, faltam mais dados nos resultados para justificar o aumento na texa de morbidade e mortalidade. Adicionalmente, qual a definição de "taxa de morbidade" para os autores ?

Introdução:

Falta uma introdução mais robusta e clara em relação ao COVID. O Contexto inclusive relacionado ao COVID é pobremente descrito.

Novamente não estão claros os objetivos do estudo, deve estar descrito o período de comparação nos objetivos.

Novamente, há a necessidade de descrição dos termos do artigo. Qual a definição de morbidade ? Favor deixar mais claro e explicitar.

Métodos:

"O consentimento informado individual e a aprovação de um comitê de ética não foram necessários, pois este é um estudo ecológico que utilizou dados extraídos de sistemas de informação de saúde pública." O consentimento informado claramente pode ser dispensado. Mas a aprovação do comitê de ética em pesquisa do projeto/trabalho é fundamental.

"Para a análise dos dados foram considerados dois períodos: os dois últimos anos pré-pandêmicos 2018 e 2019 e os dois primeiros anos pandêmicos e com maiores taxas de morbidade e mortalidade por COVID-19 no Brasil: 2020 e 2021 10." Favor deixar mais claras as datas de inclusão de pacientes por meses inclusive.

Resultados:

Os resultados estão apresentados no texto de forma muito superficial.

Discussão:

"O presente estudo tem como limitações a subnotificação de casos e óbitos no sistema SIVEP-Gripe, de onde os dados foram adquiridos, pela falta de testes e exames diagnósticos

que no começo da pandemia ainda não estavam amplamente disponíveis. A coleta de dados de casos e óbitos por SRAG no SIVEP-Gripe por todos os agentes etiológicos, impossibilitando uma avaliação de casos e óbitos separada por agente causador. Por fim, não foram utilizados dados de cor/raça, renda e escolaridade no estudo, em razão da baixa qualidade com que esses dados são preenchidos em todo o Estado do Rio de Janeiro." O grande viés é a subnotificação no SIVEP-Gripe antes da pandemia !!

Os autores não apresentaram a comparação entre vírus. Foi relacionado Influenza X COVID ? tendo em vista que estudos atuais demonstram maior morbi-mortalidade em casos de influenza do que COVID em gestantes.

Referências:

Estamos em 2024. Não há nenhuma referência de 2023 ou 2024.

O presente estudo embora descreva um assunto muito relevante, carece muito de informações mais claras, desde a definição e a descrição da morbidade até a falta de clareza e maiores informações nos resultados. Adiciono questões éticas como a falta de liberação do comitê para a realização do estudo. Por isso e tudo descrito acima no presente estudo, não recomendo o presente artigo para publicação.

